# Progress in the Identification and Design of Novel Antimicrobial Peptides Against Pathogenic Microorganisms

**DOI:** 10.1007/s12602-024-10402-4

**Published:** 2024-11-18

**Authors:** Shengwei Sun

**Affiliations:** 1 School of Engineering Sciences in Chemistry, Biotechnology and Health, Department of Fibre and Polymer Technology, KTH Royal Institute of Technology, 100 44 Stockholm, Sweden; 2 School of Engineering Sciences in Chemistry, Biotechnology and Health, Science for Life Laboratory, Tomtebodavägen 23, 171 65 Solna, Sweden

**Keywords:** Antibiotics, Antimicrobial resistance, Antimicrobial peptides, Identification and design, Bacterial pathogens

## Abstract

The occurrence and spread of antimicrobial resistance (AMR) pose a looming threat to human health around the world. Novel antibiotics are urgently needed to address the AMR crisis. In recent years, antimicrobial peptides (AMPs) have gained increasing attention as potential alternatives to conventional antibiotics due to their abundant sources, structural diversity, broad-spectrum antimicrobial activity, and ease of production. Given its significance, there has been a tremendous advancement in the research and development of AMPs. Numerous AMPs have been identified from various natural sources (e.g., plant, animal, human, microorganism) based on either well-established isolation or bioinformatic pipelines. Moreover, computer-assisted strategies (e.g., machine learning (ML) and deep learning (DL)) have emerged as a powerful and promising technology for the accurate prediction and design of new AMPs. It may overcome some of the shortcomings of traditional antibiotic discovery and contribute to the rapid development and translation of AMPs. In these cases, this review aims to appraise the latest advances in identifying and designing AMPs and their significant antimicrobial activities against a wide range of bacterial pathogens. The review also highlights the critical challenges in discovering and applying AMPs.

## Introduction

The discovery of antibiotics represents one of the most notable achievements in medical history [1]. They are extensively used to treat many diseases and save countless lives. However, due to the overuse and/or misuse of antibiotics, the occurrence of antimicrobial resistance (AMR) is now a serious and ongoing problem worldwide. Various multidrug-resistant microbial species have rapidly emerged and spread, and infections caused by drug-resistant pathogens have represented the leading cause of death in humans [2]. The World Health Organization (WHO) declared AMR one of the top three health challenges [3]. To fight against this, new antibiotics or effective antibiotic alternatives are urgently needed.

Antimicrobial peptides (AMPs) are typically a type of small peptides composed of short amino acid sequences. They are structurally diverse, with four major categories being recognized, including (i) α-helical, (ii) β-sheet, (iii) αβ, or (iv) non-αβ conformations [4]. AMPs are highly effective against bacteria, fungi, viruses, parasites, and cancer cells. They are integral to the host immune system and ubiquitous in all living organisms [5]. Unlike most conventional antibiotics, AMPs generally target the bacterial cell membrane and kill bacteria by disrupting the integrity of the membrane [6]. A growing body of studies also suggests AMPs may act on the cell wall, inhibit protein synthesis or enzyme activity, or act on nucleic acids intracellularly [7]. Thus, the possibility of developing resistance to AMPs is minimal [8]. These characteristics make AMPs serve as promising alternatives to current antibiotics. The past few decades have witnessed exponential growth in identifying novel AMPs with great potential for applications in medicine, agriculture, food, and aquaculture [9]. They have been achieved from numerous organisms, including plants, animals, humans, and microorganisms, using either traditional separation and purification methods or classical omics mining (e.g., genomics, transcriptomics, peptidomics, proteomics, and metabolomics) based on/beyond sequencing projects [10]. Modern computational design methods like machine learning (ML) and deep learning (DL) are also being implemented, accelerating the discovery of new AMPs with a broad range of targets and alternative effects [11].

**Fig. 1:**
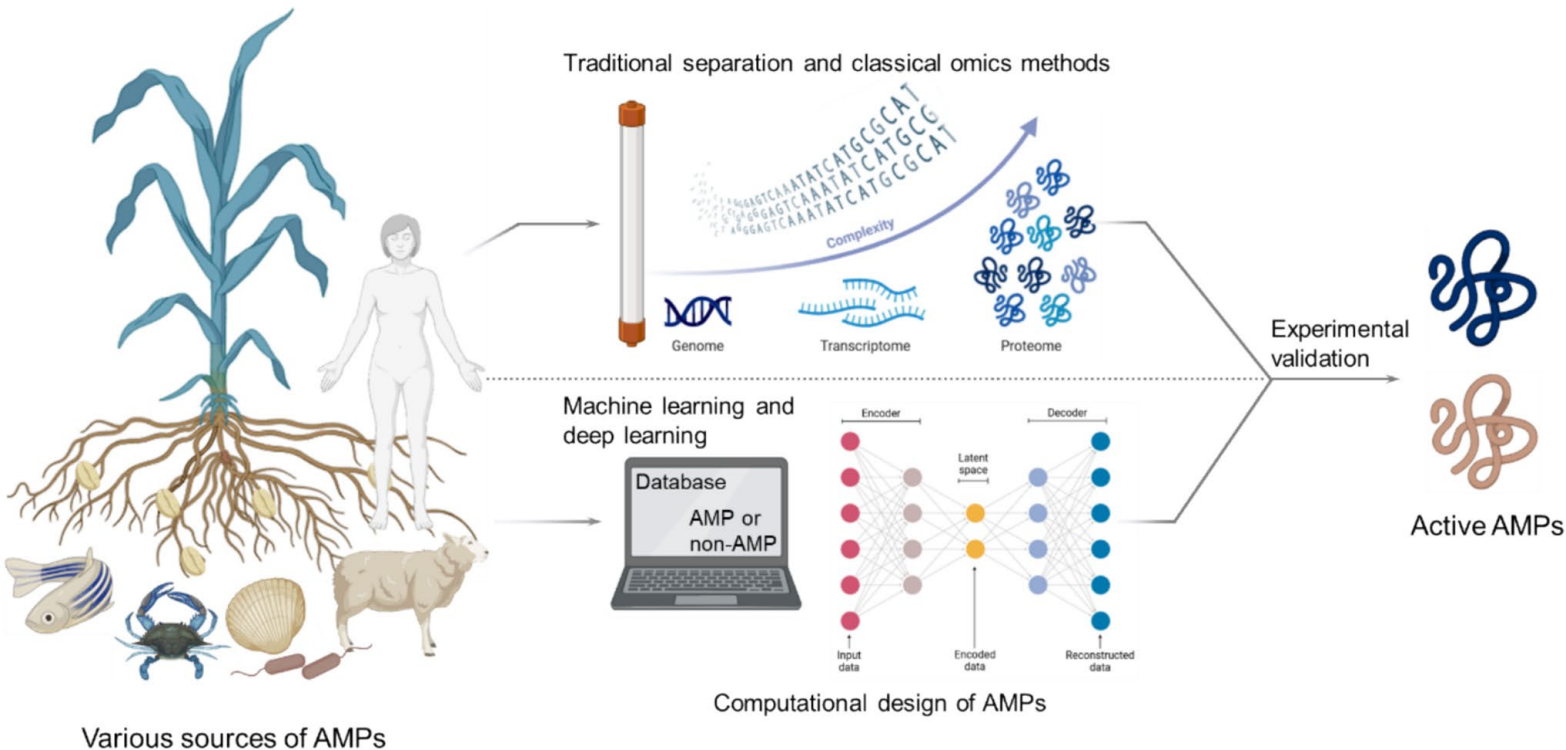
Identification and design of active AMPs from various sources. AMPs can be identified from rich natural sources (e.g., plant, animal, human, microorganism) using traditional column-based isolation and purification or bioinformatic approaches. Computer-assisted tools (e.g., machine learning, deep learning) can also be utilized to generate new AMP molecules. Subsequently, the active AMPs will be screened out using in vitro or in vivo experimental validation analyses

This review summarizes the recent advances in identifying novel AMPs from various sources (e.g., plants, animals, humans, and microorganisms) based on conventional and bioinformatic approaches. It also describes the role of computational design methods in discovering and collecting new AMPs (Fig. 1). The review further discusses the current challenges in identifying and applying AMPs. Altogether, this review provides a focused and up-to-date analysis of AMPs in response to the demands of the AMR crisis within the 2021–2024 timeframe.

## Methods for the Identification and Design of AMPs

At present, traditional column-based isolation and purification, bioinformatic approaches, and computer-assisted tools (e.g., machine learning, deep learning) are being used to generate novel AMP molecules. Conventional column separation and purification methods are mainly used for mining and identification of AMPs from protease hydrolysates or microbial fermentation media. Peptides from different sources can be extracted and purified through techniques such as solid-phase extraction, ion exchange chromatography, gel-permeation chromatography (GPC), affinity chromatography, membrane filtration, and high-performance liquid chromatography (HPLC) [12]. These techniques vary, depending on the nature of the peptide and its use. After isolation and purification of the target peptide, a number of key techniques are required to characterize the AMPs, including (1) amino acid analysis, (2) sequencing, (3) GPC, (4) sodium dodecyl sulfate–polyacrylamide gel electrophoresis (SDS-PAGE), and (5) mass spectrometry (MS). In addition, infrared spectroscopy, circular dichroism (CD), and nuclear magnetic resonance (NMR) spectroscopy have been widely used to reveal the secondary and tertiary structures of AMPs [13].

**Fig. 2:**
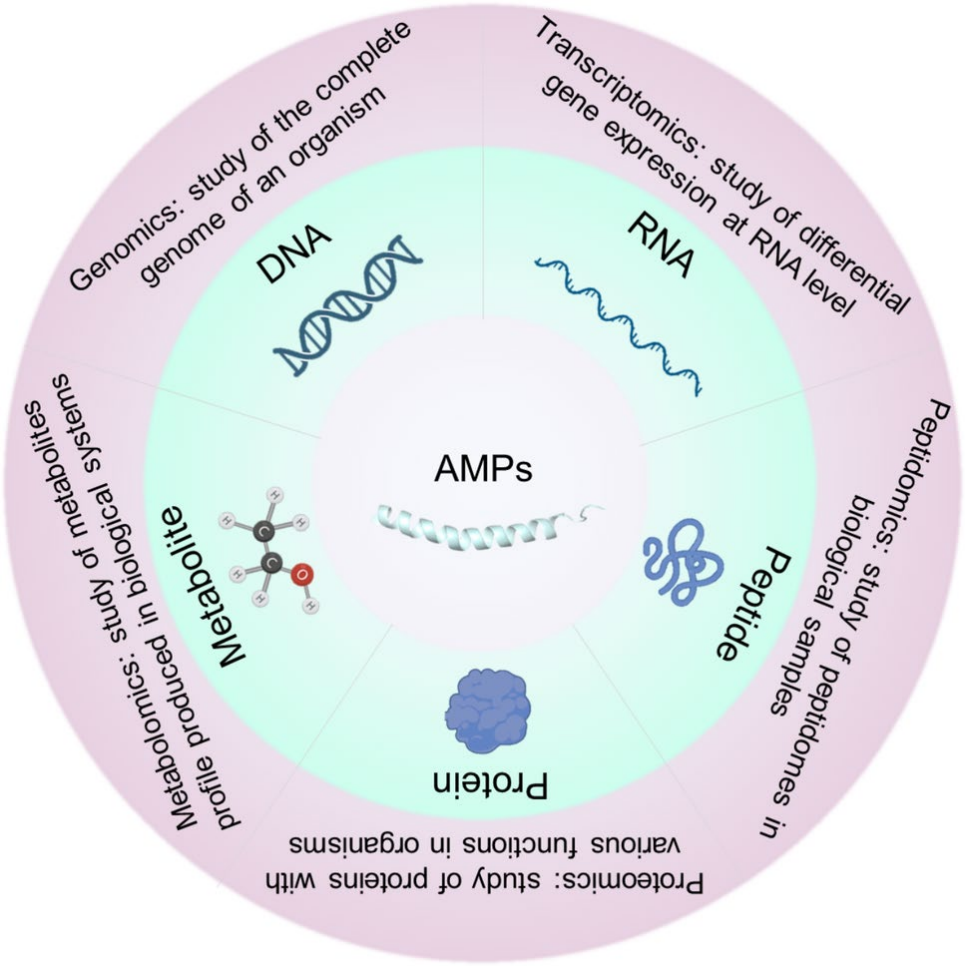
Overview of different omics approaches such as genomics, transcriptomics, peptidomics, proteomics, and metabolomics applied in the identification of AMPs in biological samples

The traditional mining methods have been considered time-consuming and laborious, showing a decline in the discovery of new AMPs. To improve the efficiency, genomics, transcriptomics, peptidomics, proteomics, and metabolomics, coupled with bioinformatics and biostatistics, have been applied to the investigation and exploration of novel AMPs (Fig. 2). Genomics is the study of the complete genome of organism by decoding the genetic code within the genetic materials (such as DNA sequence) [14]. The genome of a specific species needs to be analyzed to predict AMP coding sequences, and then bioinformatics analyses, such as domain classification and structural modeling analyses based on homology-based functional assignments and predicted peptide sequences, are used to identify candidate genomic loci encoding AMP. Comparative genomics is utilized to understand evolutionary, structural, and functional differences in AMP genes by comparing genetic information within and between organisms [15]. Recently, advances in sequencing technology and assembly algorithms have enabled the sequencing of large genomes and have provided a wealth of data for comparative genomic analysis of AMPs. Genomics studies aim to understand AMP genes at the genomic level, while transcriptomics studies determine the involvement of gene expression for specific functions [16]. In brief, it refers to the study of gene transcription and the laws of transcriptional regulation in cells. The gene encoding AMP can be investigated at the RNA (generally mRNA) level. A high-throughput sequencing platform is usually employed to sequence the transcribed mRNA. Through reverse transcribing the mRNA into cDNA, and then performing high-throughput sequencing of the resulting cDNA, a large amount of sequence data is generated [17]. The quantitative information can be further achieved by comparing the sequences to the genome or reference database for gene annotation, differential analysis, enrichment analysis, and prediction of potential new AMP genes.

In recent years, peptidomics has emerged as a research hotspot in the comprehensive qualitative and quantitative analysis of peptidomes in biological samples [18]. The peptidomics studies focus on obtaining sequences of large numbers of AMPs from HPLC and mass spectrometry (MS) data, which offers the great advantage of providing information on individual peptides, even in very complex mixtures. Combined with bioinformatics analysis (e.g., database alignment, modeling analysis, function/activity prediction), AMPs in candidate sequences are mined. Similarly, proteomics studies involve the systematic analysis of proteins that perform various functions in cells, tissues, or organs [19]. The goal of the field of proteomics is to elucidate the assembly, function, modification, interaction, and regulation of proteins, thereby providing valuable knowledge about the dynamic characterization of cellular processes. MS-based proteomics has become an important tool for identifying and quantifying numerous proteins, including these small-molecule AMPs, with excellent sensitivity and the ability to analyze complex protein samples. Lastly, metabolomics analysis aims to determine the profile of metabolites produced in biological systems and the metabolite abundance in time and space [20]. In the context of mining AMPs, metabolomics approaches can be applied in several fields. The well-known classical approach is to analyze secondary metabolites produced by soil bacteria. Some of these secondary metabolites may exhibit antimicrobial activity, and these compounds can be further identified, purified, and characterized for the next analysis.

As AMP research progresses, new advances have fueled ongoing efforts to develop computational methods for accurate AMP prediction, such as machine learning, aimed at significantly reducing the time and effort required for experimental identification. Advanced computational methods used in AMP discovery, including machine learning (ML) and deep learning (DL), are discussed in the section “Machine Learning and Deep Learning”.

## Recent Advances in the Identification of AMPs

### Plant-Derived AMPs

Plants produce an array of secondary metabolites, including antimicrobial peptides, which form an effective defense system against invasion by insects, bacteria, and fungi [21]. The composition of AMPs, even in a single plant, is quite complicated. They have a variety of structures, functions, expression patterns, and targets [22]. Previously, many plant AMPs have been identified and isolated, such as thionins, defensins, lipid transfer proteins, knottin-type peptides, α-hairpinin family peptides, and snakins [23]. Recently, the nodule-specific cysteine-rich (NCR) small peptides from legume plants have attracted increasing attention, because they have significant antimicrobial activity against human, animal, and plant bacterial and fungal pathogens. For example, 50 NCR peptides from *Cicer arietinum*, *Galega orientalis*, *M. truncatula*, *M. sativa*, and *Pisum sativum* were chemically synthesized and found to exhibit antimicrobial activity against eight human pathogens [24]. The most potent AMPs killed bacteria at different concentrations (0.8–3.1 μM). Positive net charge and high isoelectric point might be responsible for their antimicrobial activity. Moreover, experiments on the activity of shorter peptide derivatives led to the identification of key regions, which played an important role in the antimicrobial action. Similarly, three other novel NCR peptides (NCR888, NCR992, and NCR094) were identified from the legume plant, *Medicago truncatula*, and showed an inhibitory activity against the methicillin-resistant *Staphylococcus aureus* (MRSA) and *Klebsiella pneumoniae* based on in vitro and ex vivo assays (e.g., antimicrobial activity, antibiofilm activity, toxicity profiling) [25]. Notably, NCR992 and NCR094 killed *K. pneumoniae* completely at 50 and 25 µM, respectively.

Apart from these endogenous AMPs, an increasing number of AMPs identified from plant protein hydrolysates using enzymatic hydrolysis has been reported [26]. Plant seed protein represents an important source of AMPs. For example, a novel AMP, namely MOp3, was achieved using a one-step procedure combining cell membrane chromatography (CMC) and live bacteria adsorption from seed protein hydrolysates of *Moringa oleifera* and identified by liquid chromatography-tandem mass spectrometry (LC–MS/MS) [27]. MOp3 (sequence: MCNDCGA) exhibited the maximum inhibitory effect on the pathogen *S. aureus* (MIC value = 2 mg/mL). Further study suggested that MOp3 could increase cell membrane permeability and reduce the activities of intracellular AKP, LDH, and ATPase, eventually leading to irreversible membrane damage to *S. aureus* cells [28]. Moreover, cells treated with MOp3 showed ROS accumulation, nuclear rupture, and DNA fragmentation, causing cell apoptosis. Similarly, another novel AMP, MOp2 (sequence: HVLDTPLL), was identified and characterized from *Moringa oleifera* seed protein hydrolysates using LC–MS/MS and circular dichroism (CD) spectra [29]. The chemically synthesized peptide displayed a β-sheet structure and a significant inhibitory activity against *S. aureus* (MIC value = 2.204 mM). It also showed high stability, with more than 90% of antimicrobial activity maintained under 5% salt and 78% of antimicrobial activity retained at a high temperature of 115 °C for 30 min. Further mechanism study revealed that MOp2 increased the membrane permeability of *S. aureus* cells, causing irreversible damage to the cell membrane and releasing intracellular nucleotide pools.

### Animal-Derived AMPs

The ubiquitous exposure of animals to pathogens daily through physical contact, ingestion, and inhalation is well-established [30]. Over the past decade, the role of AMPs in animal innate immunity to avoid infection and protect the host has become increasingly evident. Hence, animals are a crucial source of AMP. The literature search reveals that in recent years, many AMPs have been found mainly in aquatic animals (such as fish, crabs, and shellfish) and mammals (Table 1). For instance, several active AMPs were purified from the epidermis of catfish (*Clarias batrachus*) using reverse-phase ultra-fast liquid chromatography (UFLC) and determined using matrix-assisted laser desorption/ionization-time of flight (MALDI-TOF) [31]. They showed broad-spectrum antimicrobial activity against bacterial pathogens, including multidrug-resistant clinical isolates and fungal organisms. Of these, the 25 kDa peptide had high homology to pleurocidin-like peptide (*Plp*) from a fish *Pleuronectes americanus* based on the BLAST analysis and Mascot search. A new AMP Spasin_141-165_ derived from marine crab *Scylla paramamosain* was identified based on the analysis of the constructed transcriptome database, which exhibited strong and broad-spectrum antibacterial and antifungal activity at MICs ranging from 0.75 to 48 μM [32]. It was able to kill *Acinetobacter baumannii* and *S. aureus* within 90 min and 2 h, respectively. Mechanistic studies suggested that Spasin_141-165_ changed the cell membrane permeability of bacterial pathogens, resulting in ATP release and intracellular reactive oxygen species (ROS) accumulation. Additionally, using nanoLC-ESI–MS-MS technology and bioinformatic predictions (e.g., CAMPR3, AMP scanner vr.2, iAMPpred) allowed the identification of three novel AMPs (Nv-p1, Nv-p2, and Nv-p3) from the marine mollusk *Nerita versicolor* [33]. They all adopted a random coil structure and exhibited inhibitory activity against *Pseudomonas aeruginosa*. Among them, Nv-p3 demonstrated the highest inhibitory activity at a MIC of 1.5 µg/mL in the radial diffusion assays. They also demonstrated effective antibiofilm activity against *Candida albicans*, *Candida parapsilosis*, and *Candida auris*.

**Table 1:** AMPs identified from various animal sources

Source	Method	Sequence	Activity	Mechanism	Ref
Chinese horseshoe crab	Transcriptome analysis	MCHKAMLTLVVLIALCGIMKT	A broad spectrum of antimicrobial activity against Gram-negative and Gram-positive bacteria and fungi	By directly binding to LPS, DNA, and chitin	[42]
*Mytilus coruscus*	Transcriptomic data	HYKGCPFNQHRCHVYCLSHGCKGGYCGGWFRLKCKCIGC, etc	Response to different microbes in immune-related tissues	/	[43]
African Catfish	Peptide extraction and isolation	AALKKALTAGGY, AALKKALAAGGY, FGGAGVGKTVL, etc	Exhibiting antimicrobial activity on *E. coli* and *S. aureus*	/	[44]
Sturgeon (*Acipenser ruthenus*) Spermary	Enzymatic hydrolysis and purification	NDEELNKLM and RSSKRRQ	Excellent inhibitory activity against *E. coli* with an inhibitory rate of 76.46%	Interacting with the DNA gyrase and dihydrofolate reductase of *E. coli* via salt bridges and hydrogen bonds	[45]
Fish gelatin hydrolysates	Peptidomics and bioinformatics	GPLGAAGP (P1), GRDGEP (P2), and MTGTQGEAGR (P3)	Antibacterial activity against *E. coli*, *P. aeruginosa*, *S. aureus*, and *K. pneumoniae*, and the inhibitory potential of angiotensin-converting enzyme (ACE) and dipeptidyl peptidase IV (DPP-IV)	Sufficient binding energy to inhibit both ACE and DPP-IV enzymes, with excellent three-dimensional conformation	[46]
Mandarin fish (*Siniperca chuatsi*)	Transcriptomes data	MKCTALFLVLSLV, IFHHIFKGIVHVGK, MKGLSLVLLVLLLMLAVGEG, etc	Playing a key role in mandarin fish in defense against *A. hydrophila* infection	/	[47]
European Sea Bass (*Dicentrarchus labrax*)	Whole-genome shotgun sequence	MAYYRVVALALLVVLLLNVVENEAASFPWSCPSLSGVCRKVCLPTELFFGPLGCGKGFLCCVSHFL, etc	/	/	[48]
Medaka (*Oryzias latipes*) fish	Isolation and mass spectrometric analysis	IRIILRAQGALKI	A broad-spectrum toxicity against pathogenic bacteria including drug-resistant strains	Inducing deregulation of periplasmic peptidyl-prolyl isomerases and reducing the RNA level of an upstream regulator of envelope stress responses	[49]
Golden pompano, *Trachinotus ovatus*	Comparative genomics	/	Participating in the immune response to the infection with *S. agalactiae* and *C. irritans* through the MAPK signaling pathway	/	[50]
*Hirudinaria manillensis*	Bioinformatic analysis	RKYKEKKDKSQNKKKKRKCMIL	Exhibiting good antimicrobial activity against *S. aureus*, with a MIC of 6.25 μg/mL	An obvious disruption of the plasma membrane and inhibition of biofilm formation and promotion of biofilm eradication in *S. aureus*	[51]
Chinese tubular cone snail	Multi-omics (genomics, transcriptomics, and peptidomics) data	NAPGIYARTGLFLKWIEDTIRQ, VLELAGNAARDNKKTRIIPRHLQL, RAKRRIQY, etc	Showing inhibition activity against gram-negative bacteria *E. coli* and gram-positive bacteria *S. dysgalactiae* and *M. luteus*	/	[52]
Sheep milk	*Lactobacillus plantarum* (KGL3A) fermentation and LC/MS	SPAQTLQWQVLPNAVPAK, GPFPILF, SCQDQPTAMAR, AMKPWTQPKTNAIPYVRY, IPAVFL, and KFWGKYLYEVAR	Showing antimicrobial properties against *E. coli*, *S. typhimurium*, *E. faecalis*, and *B. cereus*	/	[53]
Mare’s milk and koumiss	Peptidomics	GNYDAAQRGPGGAWAAKVIS, GNYDAAQRGPGGAWAAKVISDARES, FLKEAGQGAKDMWRA, etc	Having antimicrobial activity and significant and stable antibacterial effects	Inhibiting *S. aureus* by changing its morphology and killing it via the induction of protein leakage	[54]

“/” stands for “not available”.

Milk protein-derived AMPs have gained extensive attention owing to their broad range of antimicrobial activities, the safety of raw materials, and the slow development of AMR [34]. For example, utilizing bioinformatics techniques (including online server BIOPEP-UWM, CAMP database, APD2 database, and hidden Markov models (HMMs)) identified a novel AMP in lactoferrin of camel milk, which showed the inhibitory effect on the growth of pathogenic bacteria *A. baumannii*, *P. aeruginosa*, and *S. aureus* with MIC values of 62.5, 31.25, and 31.25 μg/mL, respectively [35]. Iram et al. obtained several AMPs from goat milk fermented with *Lactobacillus rhamnosus* C25 [36]. These AMPs were released through proteolysis of the goat milk proteins with proteolytic enzymes from *Lactobacillus* strains. The peptide fractions were collected using ultrafiltration with different-sized MWCO (< 10, < 5, and < 3 kDa) membranes and analyzed using RP-HPLC and LC–MS/MS. In silico analysis indicated that most of these AMPs (< 5 kDa) showed maximum antibacterial activity against *E. coli*, *E. faecalis*, and *S. typhi* and natural cationic properties, which was an important factor for the interaction with the bacterial negative charge membrane. Among these, AMPs (IGHFKLIFSLLRV and KSFCPAPVAPPPPT) exhibited low ligand binding energy of − 7.5 and − 7.6 kcal/mol, respectively, for MurD, a Mur ligase capable of regulating peptidoglycan (PG) biosynthesis in order to maintain the relative thickness of the PG layer in Gram-negative bacteria [37].

Besides, newly identified AMPs from spiders, *Monochamus alternatus*, and earthworms indicate that the animal kingdom equipped with versatile self-defense mechanisms is a rich source of natural AMPs. Specifically, a novel AMP, namely, Lycotoxin-Pa2a, with potent antibacterial activity was identified from the spider venom gland transcriptome library of the spider *Pardosa astrigera* through a homologous sequence search [38]. Structure prediction revealed that the peptide formed antiparallel β-strand and α-helix at both N-terminus and C-terminus, which were commonly found in toxin peptides. Lycotoxin-Pa2a (Lytx-Pa2a) showed comparable or even stronger antibacterial activity than melittin against pathogens based on colony formation and MIC assays. Mechanism studies suggested that Lytx-Pa2a could disrupt both the cell’s inner and outer membranes and induce ROS accumulation simultaneously. Han et al. isolated and identified a novel knottin-type AMP derived from *Monochamus alternatus* based on the transcriptome data [39]. The knottin peptide (MaK) was composed of 34 amino acids, of which 11 amino acid residues were in an extended strand conformation, 4 amino acid residues formed a β-turn, and 19 amino acid residues were in a randomly coiled configuration. It showed superior stability, low toxicity, and the capacity to suppress pine wood nematodes. Particularly, MaK exhibited significant antibacterial activity with a MIC of 36.29 µg/mL against *Bacillus thuringiensis* and *E. coli* and 72.58 µg/mL against *Serratia marcescens*. Additionally, a novel AMP, named EWAMP-R (RIWWSGGWRRWRW), was identified based on the prediction and sequence optimization of potential AMPs from the open reading frames (ORFs) in the *Eisenia andrei* genome [40]. A number of ORFs were read and translated into amino acid sequences using NCBI ORFfinder, and the potential AMPs were predicted using CAMP_R3_ [41]. As a result, EWAMP-R demonstrated excellent antimicrobial activity against various bacteria, including drug-resistant *E. coli* and *S. aureus* (MIC of 16 μg/mL and 16 μg/mL, respectively), by disrupting bacterial cell membrane integrity. This study demonstrated that earthworms served as a new source of antibacterial agents in response to the AMR issue. More examples of newly identified AMPs from animal sources are shown in Table 1.

### Human-Derived AMPs

Human-derived AMPs protect humans from microbial infections through a variety of mechanisms [55]. Various types of AMPs, such as defensins [56], granulysins [57], histatins [58], thrombocidins [59], and cathelicidins [60], are produced in humans. They have been identified in many tissues or surfaces such as the eyes, skin, ears, mouth, lungs, intestines, and urinary tract. Notably, the 37-residue LL37 (sequence: LLGDFFRKSKEKIGKEFKRIVQRIKDFLRNLVPRTES) is the only recognized AMP in humans and has been the focus of research for the past several decades [61–64]. It has broad-spectrum antibacterial and anti-biofilm activity. The host defense activity of LL37 is manifested in various parts of the body, ranging from epithelial cells in various organs (testis, skin, respiratory and gastrointestinal tracts) to immune cells. In addition to its typical antimicrobial activity, LL37 exerts several other host defense activities, including modulation of the inflammatory response, chemoattraction, and wound healing at the site of infection. Currently, the antimicrobial peptide LL-37 is recognized as a potential alternative to traditional antibiotics. However, despite its potent antimicrobial properties, LL-37 has several limitations, including high cost in production, low activity in physiological environments, susceptibility to proteolytic degradation, and high toxicity to human cells, and thus has not yet received regulatory approval as a peptide antibiotic [65].

Also, human milk feeding can decrease the risk of necrotizing enterocolitis (NEC), partly because of the bioactive peptides released from milk proteins during digestion [66]. For instance, Lyu et al. applied mass spectrometry–based peptidomics to identify bioactive peptides with antimicrobial activity in colostrum before and after in vitro digestion [67]. A homology search of all identified peptides against the Milk Bioactive Peptide Database revealed that 121 peptides in the undigested samples showed more than 80% homology with known bioactive peptides, and 64 peptides in the digested samples showed more than 80% homology with known bioactive peptides. Among them, four peptides (αS1-casein_157-163_, αS1-casein_157-165_, β-casein_153-159_, and plasminogen_591-597_) were found to exhibit antimicrobial activity against common pathogenic bacteria, including *Klebsiella aerogenes*, *Citrobacter freundii*, and *Serratia marcescens*. This study provided important insights into the potential of breast milk–derived AMPs to inhibit the growth of bacterial pathogens in the infant’s gut and prevent infection.

The human gut is home to a diverse and dynamic microbiota that is essential for digestion, metabolism, and immune development and has a critical impact on human health [68]. The intestinal epithelium constitutes the primary defense barrier and produces AMPs that may act as innate immune effectors to modulate the gut microbiota by directly killing or inhibiting microorganisms. In addition, intestinal AMP may act synergistically with other gut microbiota and antimicrobial agents to maintain intestinal homeostasis by combating multi-antibiotic-resistant (MAR) bacteria [69]. Understanding the complex interactions between AMP and the gut microbiota is critical to developing strategies to enhance immune responses and combat gut microbiota infections. Current research is continuing to reveal new aspects of this complex relationship, deepening our understanding of the factors that influence gut health.

### Microorganism-Derived AMPs

Microbial AMPs have been recognized as a rich source of antimicrobial agents. There are two types of microorganism-derived AMPs: ribosomally synthesized peptides (e.g., bacteriocins) and non-ribosomally synthesized peptides (e.g., lipopeptides, glycopeptides, cyclopeptides, lipoglycopeptides). There have been increased reports of novel AMPs derived from microbial sources with great potential as alternatives to existing small-molecule antibiotics. Their antimicrobial activity, biological processes (biosynthesis, transport, self-immunity), action mechanisms, and a wide range of applications have been extensively reviewed in previous studies [70–72]. Recently, the growing accessibility to omics techniques (such as metagenomic and meta-transcriptomic) has provided more opportunities to identify new microbial AMPs [70]. For example, two AMPs, namely HG2 and HG4, were identified from a rumen microbiome metagenomic dataset [73]. Specifically, the original library dataset containing 2,547,270 predicted protein sequences was downloaded from the website (http://portal.nersc.gov/project/jgimg/CowRumenRawData/submission/). Other datasets including known AMPs and synthetic AMPs were used for similarity analysis prediction/identification of novel AMP candidates based on the MATLAB toolbox Gait-CAD and its successor SciXMiner. AMPs HG2 and HG4 identified from the 829 sequences were the most active peptide against multidrug-resistant (MDR) bacteria, especially methicillin-resistant *Staphylococcus aureus* (MRSA) strains. They also exhibited in vitro anti-biofilm and anti-inflammatory activities and showed little toxicity to human primary cell lines. To identify potential AMPs from a rumen eukaryotic meta-transcriptomic sequence, 208 potential AMPs were used to build a screening library [74]. Thirteen of these 208 peptides exhibited promising activity during the spot high-throughput antimicrobial activity screening. Of these, an α-helical peptide named Lubelisin (GIVAWFWRLAR) was identified with potent antimicrobial activity against MRSA. It could kill MRSA USA300 and EMRSA-15 strains with ≥ 10^3^ and 10^4^ CFU/mL reduction in viable cells within 30 min, respectively.

Moreover, Song et al. isolated and identified a number of lactic acid bacteria–produced AMPs in Chinese pickles using the LC–MS/MS method [75]. A novel AMP (NQGPLGNAHR) exhibited significant antibacterial activity against *S. aureus* (IC_50_ value = 0.957 mg/mL), *L. monocytogenes* (IC_50_ value = 3.035 mg/mL), *B. cereus* (IC_50_ value = 2.110 mg/mL), *B. subtilis* (IC_50_ value = 4.311 mg/mL), and *E. coli* (IC_50_ value = 3.549 mg/mL). A docking study revealed that the peptide could tightly bind to dihydrofolate reductase and DNA gyrase via hydrogen bond interactions, salt bridge formation, and metal contact, indicating that the inhibitory effect of the peptide on the growth of *S. aureus* might be mediated through the interaction between this peptide and these enzymes. Similarly, Feng et al. identified and characterized three AMPs consisting of fatty acids, 2,4-diaminobutyric acid, and amino acid structures using LC–MS/MS, which were produced by *Paenibacillus ehimensis* HD [76]. Circular dichroism (CD) showed that these peptides were mainly composed of randomly coiled and β-folded structures. They exhibited low hemolytic activity and significant antimicrobial activity against a wide range of food pathogens and fungi. Wiman et al. synthesized and screened a small library of bacteriocin Plantaricin NC8 αβ-derived lipopeptides based on the activity prediction using the servers AntiBP (http://www.imtech.res.in/raghava/antibp/) and ADAM (http://bioinformatics.cs.ntou.edu.tw/ADAM). They showed significantly improved antimicrobial activity towards both gram-negative and gram-positive bacteria, including the critical multidrug-resistant ESKAPE pathogens [77]. These lipopeptides were composed of 16 amino acids terminating in fatty acid chains. They could assemble into micelles that penetrated cell membranes to inhibit and kill bacteria effectively.

It has been known for decades that microorganisms living in saline environments are producers of many valuable bioproducts, including AMP. Lach et al. identified three peptides (P1, P2, and P3) in eight metagenomes from high-saline environments, which showed robust antimicrobial activity against multidrug-resistant human pathogens [78]. The P1 peptide inhibited the *E. faecalis* (MIC_50_ of 32 µg/mL), and the P3 peptide showed inhibitory effects on both *E. faecalis* and *S. aureus* strains (MIC_50_ of 32 µg/mL). More recently, Cheng et al. have identified new aminovinyl-(methyl)cysteine (Avi(Me)Cys)-containing peptides from more than 50,000 bacterial genomes through a biosynthetic rule-based omics mining [79]. Through sequence similarity network (SSN) and sequence logo analysis, a class V lanthipeptide, massatide A, was identified with broad antibacterial activity against a wide spectrum of gram-positive pathogens, including drug-resistant clinical isolates like methicillin-resistant and linezolid-resistant *S. aureus* (MIC of 0.25 μg/mL)*.* Megaw et al. developed a new strategy for discovering AMPs based on metagenomic sequences collected from 66 soil samples [80]. The new method identified several functional AMPs that exhibited antimicrobial activity against various bacterial species. Structural modifications, including peptide extensions, showed that activity peak was observed at 9–12aa, and in most cases, the addition of the C-terminal amide could enhance the activity. This metagenomics-based strategy differed from previous approaches because it did not rely on the sequence homology of known AMPs in databases. It might provide more important opportunities to identify new AMPs of clinical relevance.

## Recent Advances in the Computational Design of AMPs

### Machine Learning and Deep Learning

Experimental methods for discovering and identifying new AMPs have been considered time-consuming and costly. Recently, computational methods, including machine learning (ML) and deep learning (DL), have been of tremendous interest in AMP discovery [11]. Typical ML involves multiple sequential steps including data pre-processing, feature extraction, intelligent feature selection, learning, and classification. Advances in DL make it possible to automatically learn the set of features for multiple tasks at the same time [81]. Predicting novel AMPs from genome sequences of various organisms can accelerate the discovery of new AMPs. The Antimicrobial Peptide Database (APD) (https://aps.unmc.edu/database/anti) also provides an empirical peptide prediction program that contributes to the testing of machine learning algorithms [82]. The ML and DL approaches effectively identify previously unknown patterns from sequences and predict the antimicrobial potential of new sequences. According to the prediction results/scores, candidate sequences will be selected, synthesized, and prioritized for experimental testing, significantly reducing the time and cost of discovering newly active AMPs. Currently, most of the activity prediction models are based on the primary structure of AMPs, and structural parameters such as amino acid composition, net peptide charge, hydrophobicity, amphiphilicity, and helix are crucial for the activity of AMPs. There are strong correlations between these parameters, and feature extraction for AMP is an important step in data analysis and machine learning. Using the various database resources and AI (such as Database of Antimicrobial Activity and Structure of Peptides (DBAASP) [83] and AlphaFold [84]) allows the efficient incorporation of activity or structure information into the computational design. With positive and negative datasets used for training and through discrimination and generation approaches, a number of novel AMP molecules with potent antimicrobial activity can be generated.

**Fig. 3:**
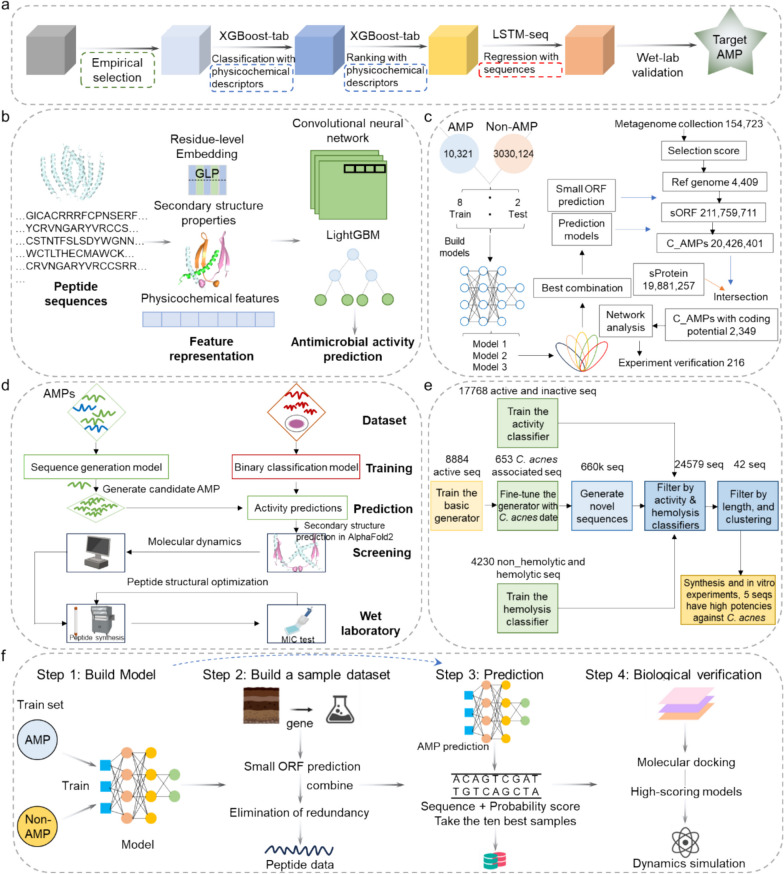
Schematic representation of ML and DL-based AMP design workflow. **a** The whole process of SMEP screening method of AMPs (Reprinted and adapted with permission from [85]. Copyright 2023, Springer Nature). **b** A predictive framework ensembles deep learning and statistical learning methods to screen small peptides with antimicrobial activity (Reproduced and adapted with permission from [86]. Copyright 2024, Springer Nature). **c** Schematic representation of candidate AMP identification from human gut microbiome data based on multiple natural language processing neural network models, including LSTM, Attention, and BERT. (Reprinted and adapted with permission from [89]. Copyright 2022, Springer Nature). **d** Design of AMPs using deep learning and molecular dynamic simulations (Reprinted and adapted with permission from [93]. Copyright 2023, OXFORD ACADEMIC). **e** A design pipeline trained on publicly available data, with generators and classifiers, using transfer learning and pre-trained protein embeddings (Reproduced and adapted with permission from [94]. Copyright 2024, Springer Nature). **f** The workflow of a combined AMP screening model based on LSTM neural networks with an attention mechanism (Reprinted and adapted with permission from [95]. Copyright 2024, MDPI)

For example, Huang et al. reported a machine-learning process to identify valid AMPs from a virtual peptide library of hundreds and billions of sequences of 6–9 amino acids [85]. In brief, a sequential model ensemble pipeline with multiple steps (e.g., empirical selection, classification, ranking, regression, wet-lab validation) was proposed (Fig. 3a). At the beginning, the least promising peptides in the search space were removed in the pre-filtering process, called empirical selection. Subsequently, by sequentially assembling multiple functional modules in the appropriate order, a series of coarse filters were used to “pool” the potentially optimal peptide sequence space into precise modules for MIC prediction. The necessity of each module in the process was confirmed through wet-lab validation. The funnel-like machine learning process design allowed the quick and accurate discovery of valid AMPs from the entire hexapeptide, heptapeptide, octapeptide, and nonapeptide search space. Notably, 15 candidate sequences were identified, synthesized, and experimentally tested from a pool of 512 billion sequences in 27 days. Three antimicrobial hexapeptides were finally identified and exhibited potent inhibitory activity against multiple multidrug-resistant pathogens from clinical isolates. When applied to mice suffering from bacterial pneumonia, AMP aerosolized formulations demonstrated remarkable therapeutic efficacy comparable to penicillin, negligible toxicity, and a relatively low tendency to induce the development of resistance. Zhong et al. proposed a prediction framework that combined deep learning and statistical learning methods to screen potential AMPs [86]. This method integrated multiple LightGBM classifiers and convolutional neural networks (CNNs) that utilized a variety of predicted sequence, structural, and physicochemical properties from residue sequences extracted through various machine learning paradigms (Fig. 3b). Specifically, three sequence deep representation learning embedding methods (SSA, UniRep, and ESM-1b) were used to extract sequence embedding features. A deep learning–based prediction tool, NetSurfP-3.0, provided estimates of relative surface accessibility and α-helix, β-strand, and coil secondary structure element probabilities for peptide residues [87]. The machine learning–based SVM-Prot protein functional family prediction framework generated physicochemical features for each peptide and organized them into 188-dimensional vectors. Subsequently, the representations were divided into four groups and fed into basic classifiers (including LightGBM and CNN), followed by a fully connected layer that collected the results of the LightGBM predictor and combined the discriminative power of the CNN with weight selection techniques. Comparative experiments showed that the method outperformed other state-of-the-art methods regarding representativeness metrics on independent test datasets. Wani et al. investigated various machine learning classifiers to build computational models for the prediction of AMP based on a large diversity of AMPs (2638) and non-AMPs (3700) from various online databases (APD3, CAMP_R3_, and LAMP), including Decision Trees (DTs), Ensemble Learning, Naive Bayes (NB), k-Nearest Neighbors (k-NN), Quadratic Discriminative Analysis (QDA), random forests (RF), and support vector machines (SVMs) [88]. Molecular descriptors for the peptide sequences contained in the dataset were calculated using the open-source cheminformatics toolkit PyDPI-1.0, while the data normalization was carried out using the MinMaxScaler method of Scikit-learn to scale the data in a range of 0 to1. For splitting the dataset into training and test data, the StratifiedShuffleSplit method was implemented. Moreover, for the model development, an open-source ML learning toolkit named Scikit-learn was used. These developed models were subsequently assessed by a fivefold cross-validation method. The results revealed that the RF classifier-based model performed best in both internal (sensitivity: 90.05%, accuracy: 91.40%, precision: 89.37%, specificity: 92.36%) and external validation (sensitivity: 85.21%, accuracy: 89.43%, precision: 88.92%, specificity: 92.43%). This might be due to the fact that ChargeD2001, PAAC12, and Polarity T13 performed best in the RF classifier, and these features contributed to the identification of the antimicrobial potential of the peptides. In addition, the RF classifier-based model correctly predicted known AMPs and non-AMPs, and these were retained for additional external validation sets.

Ma et al. identified several AMP candidates from human gut microbiome data using different natural language processing neural network models, such as long short-term memory (LSTM), attention mechanism, and bidirectional encoder representations from transformers (BERT) [89]. In terms of the data collection, 10,327 AMP sequences with lengths ranging from 6 to 518 AAs and 3,029,894 non-AMP sequences were used as training and test datasets to build a model (Fig. 3c). Multiple metaproteome and large-scale metagenome datasets were employed to ensure that the small open-reading frames (sORFs) were indeed expressed and identify the AMPs that had the inhibitory potential, respectively. Candidate AMPs were predicted using the EMBOSS software package (version 6.6.0.0) representative species-level genomes [90]. A total of 181 of the 2349 sequences were identified as active AMPs exhibiting efficient antimicrobial activity (positive rate > 83%). Further characterization of the 11 most potent AMPs demonstrated that they were highly inhibitory against drug-resistant gram-negative pathogens and could reduce bacterial loads by more than tenfold in a mouse model of bacterial lung infection. Determining the three-dimensional structure of peptides is critical to understanding their structure–activity relationships and to designing more efficient analogs [91]. AlphaFold2 uses deep learning to efficiently and accurately predict protein structures, a major breakthrough in artificial intelligence [92]. Cao et al. combined several natural language processing models to design and recognize AMPs, including Sequence Generation Adversarial Networks (SeqGAN), BERT, and Multilayer Perceptron (MLP) [93]. Briefly, after preprocessing and dataset splitting within the collected AMPs and non-AMPs, the SeqGAN model was used to generate novel AMPs, followed by the BERT model and MLP to construct an AMP classification model. To pick up candidate AMPs, the AMP structures were predicted by AlphaFold2 and the stability of the peptides was assessed by MD simulations (Fig. 3d). In the wet laboratory, peptides with high stability were chemically synthesized and characterized. Six potential AMPs were finally screened, which belonged to a novel class of AMPs with low homology to known AMPs. During the preliminary bioactivity testing, one of the peptides, A-222, was found to be inhibitory to gram-positive and gram-negative bacteria, with MIC values of 16 μg/mL against *B. subtilis* 168 and *L. enzymogenes* YC36, as well as MIC values of 32 and 64 μg/mL against *S. maltophilia* WH 006 and *P. aeruginosa* PAO1, respectively.

Dong et al. utilized a deep learning pipeline to screen peptides with inhibitory activity against *Cutibacterium acnes* [94]. In the model, 8884 active AMPs collected from the Database of Antimicrobial Activity and Structure of Peptides (DBAASP) were used as training (75%) and testing (25%) datasets [83]. Furthermore, a subset of 653 *C. acnes*-related AMPs was used for fine-tuning toward *C. acnes*-specific AMPs (Fig. 3e). The generators and classifiers used transfer learning and pre-trained protein embeddings and trained on public data. A phylogenetic tree was also constructed to enhance the training data targeting *C. acnes*. To pursue the optical AMPs, the peptide length was taken into consideration based on several guiding principles. Among these, 10 amino acids as the minimal peptide length were used as a delineation line to ensure the typical alpha-helical structures these peptides adopted to maintain their efficacy. Finally, a group of 42 new peptides was then selected, synthesized, and experimentally validated for their antimicrobial potencies. Five exhibited high activity and selectivity against *C. acnes* with MICs ranging from 2 to 4 µg/mL. Liu et al. proposed a conjunctive screening model based on an LSTM neural network and an attention mechanism to accurately identify potential AMPs by analyzing the peptide profiles of enzymatic hydrolysis simulation products of proteins expressed in the genomes of sludge microorganisms [95]. The training dataset was made up of 4370 positive samples (AMPs) and 5132 negative samples (non-AMPs), while 2211 positive samples and 2543 negative samples were used as validation datasets (Fig. 3f). The attention-based LSTM (ALSTM) model was used to process sequence data and extract key features. Based on a combination of LSTM and ALSTM networks to form a composite model, the prediction framework was generated for the recognition of AMPs. Three potential AMP candidates, LLPRLLARRY, FRTTLAPHVLTRLLAPCW, and GVREIHGLNPGGCLHTVRLVCR, were validated using molecular docking and dynamic simulation, which exhibited high specificity to target proteins, confirming that the proposed AMP screening model was valid. Although existing computational methods have achieved satisfactory performance, there is still much room for model improvement. Many efforts have been made over the past 3 years to optimize and integrate computational methods in the discovery of highly potent AMPs. More examples of this are shown in Table 2.

**Table 2:** Machine learning and deep learning for the identification of AMPs

Model	Method	Result	Access website	Ref
AMPSphere	Incorporating c_AMPs predicted with ML using Macrel, a pipeline that uses random forests to predict AMPs	A total of 79 peptides among 100 synthesized and tested AMPs are active, with 63 targeting pathogens in vitro and in vivo	https://ampsphere.big-data-biology.org/	[96]
UniproLcad	Using deep learning networks encompassing the bidirectional long and short memory network (Bi-LSTM), one-dimensional convolutional neural networks (1D-CNN), and an attention mechanism	Competitiveness in the field of AMP identification through tenfold cross-validation and independent testing	https://github.com/harkic/UniproLcad	[97]
HydrAMP	A cVAE-based model, which is specifically trained to perform analogue generation both from positives and negatives and unconstrained generation	The high activity of nine peptides produced as analogs of clinically relevant prototypes	https://hydramp.mimuw.edu.pl	[98]
TriStack	A machine learning–based module using a multi-layer residual network	Outperforming all leading methods in both AMPs and anti-inflammatory peptide (AIP) prediction	https://github.com/hjy23/TriStack	[99]
E-CLEAP	Based on the Multilayer Perceptron Classifier (MLP Classifier)	Achieving accuracies of 97.33% and 84% for the amino acid composition (AAC) and pseudo amino acid composition (PseAAC) features, respectively	https://github.com/Wangsicheng52/E-CLEAP	[100]
iAMP-DL	Constructed using two well-known deep learning architectures: the long short-term memory architecture and convolutional neural networks	An effective, robust, and stable framework for detecting promising short AMPs	https://github.com/mldlproject/2022-iAMP-DL	[101]
AMPlify	An attentive deep learning model that improves computer-simulated AMP prediction by applying two attentional mechanisms on a bi-directional long short-term memory (Bi-LSTM) layer	Four novel AMPs against a wide range of bacteria, including carbapenemase-resistant *E. coli* isolates	https://github.com/bcgsc/AMPlify	[102]
AVPIden	A machine learning–based double-stage scheme to identify AMPs	Convincible performance to predict AVPs and their targets	http://awi.cuhk.edu.cn/AVPIden/	[103]
AMPGAN v2	A bidirectional conditional generative adversarial network (BiCGAN)-based approach for rational AMP design	Novel, diverse, and customized AMPs for specific applications, making the model an efficient AMP design tool	https://gitlab.com/vail-uvm/amp-gan	[104]

### De Novo Design

**Fig. 4:**
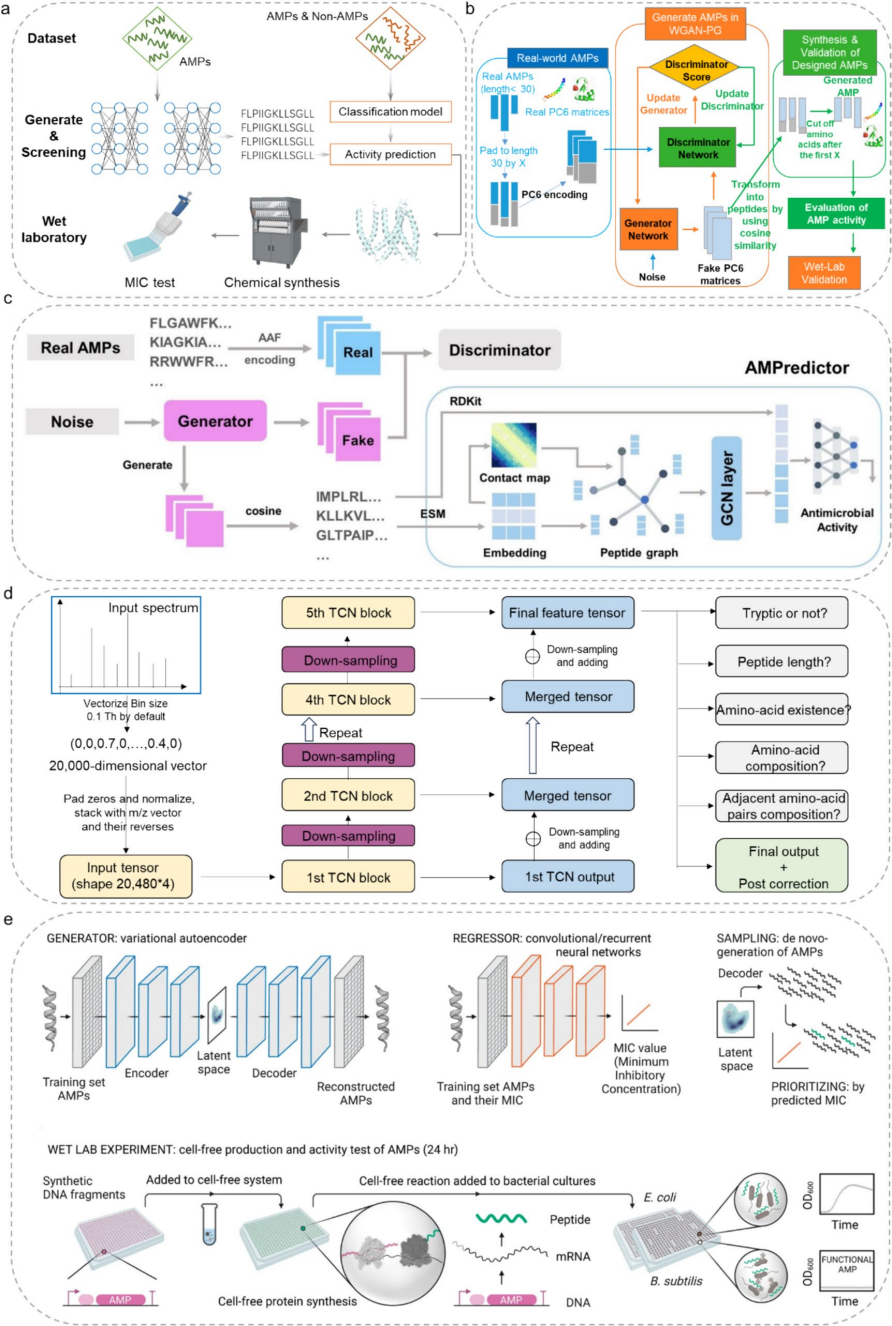
Workflow of the de novo development of AMPs. **a** The workflow of the AP_Gen algorithm. (Reprinted and adapted with permission from [105]. Copyright 2024, Springer Nature). **b**. The workflow of training WGAN-PG to generate AMPs with filters of assessment on AMP activity in silico. (Reproduced and adapted with permission from [106]. Copyright 2023, MDPI). **c** The overall framework of GAN generator and AMPredictor. (Adapted with permission from [108]. Copyright 2024, eLife). **d** The Neural Network Architecture of PepNet. (Reprinted and adapted with permission from [109]. Copyright 2023, Springer Nature). **e** The workflow for de novo development of AMPs through deep learning and cell-free biosynthesis. (Adapted with permission from [112]. Copyright 2023, Springer Nature)

Efficient and precise AMP design is increasingly applied in the research and development of AMP. The computational de novo design offers a great opportunity to address several challenges in new AMP discovery. Peptide sequences of a certain length can be obtained by randomizing the 20 natural amino acids, and then screening potential AMPs with machine learning is an effective method to obtain novel AMPs. For example, Yin et al. developed a new machine learning–assisted AMP prediction model, AP_Sin, which was trained based on 1160 AMP and 1160 non-AMP sequences to computationally de novo design novel AMPs [105]. It was established with a peptide sequence generator, AP_Gen, based on recombinant dominant amino acid and dipeptide composition (Fig. 4a). Inputting the parameters of 71 tridecapeptides from the Antimicrobial Peptide Database (APD) into AP_Gen randomly generated a library containing 17,496 de novo designed tridecapeptide sequences, from which AP_Sin screened out 2675 candidate AMP sequences. The 180 AMP sequences were randomly selected and then chemically synthesized. There were 18 peptides with significant antimicrobial activity against various tested bacterial pathogens and 16 with inhibitory capacity to at least one pathogenic strain (MICs < 10 μg/mL). Lin et al. de novo designed several novel AMPs based on training a Wasserstein generative adversarial network with gradient penalty (WGAN-GP) [106]. A total of 3195 AMPs with less than 30 amino acids were chosen and encoded by PC6 (Fig. 4b). A discriminator and a generator in the GAN model were used to learn features from real data and create data that resemble real AMPs, respectively. The AMPs generated from GAN were based on DCGAN, a convolutional network-based GAN [107]. The quality of the GAN-designed peptides was further assessed by computer simulations, where eight peptides (called GAN-pep 1–8) were chosen by the AMP intelligent classifier and chemically synthesized for subsequent activity assay. Seven out of eight GAN-designed peptides showed potent antimicrobial effects. In addition, GAN-pep 3 and GAN-pep 8 exhibited broad-spectrum antimicrobial activities against antibiotic-resistant strains, such as carbapenem-resistant *P. aeruginosa* and methicillin-resistant *S. aureus*.

Until now, more than 120 AMPs have been identified by deep learning models in the past 3 years; however, the efficacy of most of these AMPs has not been yet reflected in clinical trials. To this end, Dong et al. developed a computational framework for de novo AMP design by linking a deep generative module with a graph-encoding activity regressor (AMPredictor) [108]. The generative adversarial network (GAN) module was trained with 3280 AMP sequences and their MICs to capture the pattern of AMPs (Fig. 4c). A generator network and a discriminator in the GAN model were used to produce novel AMP sequences. A regression model for the task of MIC predictions was effectively trained using the graph convolution network (GCN) of the AMPredictor. Three bifunctional AMPs active both against bacteria and viruses were identified. Their ability to inhibit a wide range of pathogens was validated by experiments in vitro and animal models. Excitingly, P076 possessed a potent bacteriostatic activity with a MIC of 0.21 μM against multidrug-resistant *A. baumannii*, and P002 could broadly inhibit five enveloped viruses. This study provided a feasible approach to discovering sequences encoding both antibacterial and antiviral activity, thereby enhancing the functional spectrum of AMPs against multidrug-resistant infections. Liu et al. proposed PepNet, a fully convolutional neural network for de novo design peptide sequences at a high level of precision [109]. The temporal convolutional network (TCN) blocks-based PepNet was expected to capture the potential associations between distant peaks in the MS/MS spectra and maintain a low computational cost. Regarding the loss functions and auxiliary tasks, the average cross-entropy on non-padding positions was used as the major part of the loss, whereas monitoring the average accuracy on non-padding positions was used to choose the optimal model. PepNet utilized MS/MS spectra (high-dimensional vectors) as inputs and produced the optimal peptide sequences and their confidence scores (Fig. 4d). It was trained based on 3 million high-energy collisional dissociation MS/MS spectra from multiple human peptide libraries. The evaluation results suggested that PepNet outperformed the best-performing de novo sequencing algorithms (e.g., PointNovo [110] and DeepNovo [111]) in both position-level and peptide-level accuracy, which might serve as a complementary tool to database search engines for AMP identification in proteomics. Pandi et al. established a process that combined cell-free biosynthesis with deep learning to rapidly and cheaply produce AMPs directly from DNA templates [112]. The Giant Repository of AMP Activities (GRAMPA) which combined sequence and activity data from several public AMP databases provided validated AMP sequences including 6760 unique sequences and 51,345 total MIC measurements (Fig. 4e). The UniProtKB containing 10,612 unique non-AMP sequences was used as the non-AMP dataset. Subsequently, the generator variational autoencoder (VAE) composed of an encoder, a latent vector, and a decoder was employed for de novo AMP design. They designed thousands of de novo AMPs through deep learning to test this approach. Then, the convolutional neural network (CNN) and recurrent neural network (RNN) regressors were used to train the pooled AMP and non-AMP data. Using a computational approach, they prioritized 30 functional AMPs out of 500 candidates. Notably, six de novo AMPs had broad-spectrum activity against multidrug-resistant pathogens and did not develop bacterial resistance. Lastly, Bolatchiev, Baturin [113] performed the combinatorial de novo AMP design using LSTM recurrent neural network (RNN). The dataset containing 3100 sequences with a median length of 29 amino acid residues was used as final training data. Two hundred sequences as the number of sequences were extracted from the model after training, but two of these sequences were shorter than seven amino acid residues and were therefore discarded. The generated 198 sequences were then evaluated using the CAMP AMP prediction tool [114], and their antimicrobial activity was predicted by three algorithms including SVM, random forest, and discriminant analysis. Among various newly designed peptides, the PEP-38 and PEP-137 showed strong in vitro inhibitory activity against carbapenem-resistant isolates of *K. pneumoniae*, *Klebsiella aerogenes*, and *P. aeruginosa*.

### Current Challenges in the Identification and Application of AMPs

With the increasing rise of antibiotic resistance, AMPs have attracted much attention because of their broad-spectrum antimicrobial effects and unique action mechanisms. Many novel AMPs have been identified in recent years based on either traditional isolation and purification methods or a combination of omics and bioinformatics strategies. Compared to the conventional experimental procedure, the emergence of omics/bioinformatics has undeniably benefited the AMP discovery over the past decades, becoming an indispensable tool in developing and optimizing AMPs [115]. However, bioinformatics has evolved over decades to develop sequence-to-gene-to-function pipelines that are not necessarily suitable for discovering novel bioactive peptides in short sequences [116]. Recently, the field of computational design has brought forth various remarkable advancements in identifying novel AMPs. Unquestionably, the application of ML and DL methods can significantly accelerate the discovery and design of AMP, creating time and cost savings [11]. However, the computational design of new active AMPs still faces several challenges. For example, insufficient available AMP data inputs often lead to inaccurate computational results, which require subsequent chemical synthesis of many potential AMP candidates and experimental screening and validation. The second is that computational design requires continuous model optimization to achieve considerable reproducibility, which may require a highly trained and skilled person with specialized knowledge and capabilities to fulfill their duties. This process is still difficult for most researchers who lack the relevant expertise. Additionally, current computational methods are almost exclusively based on a binary classification of linear AMPs, i.e., AMPs and non-AMPs. This tends to ignore the natural activities of the peptides, such as antioxidant activity, anti-inflammatory activity, and antiviral activity, leading to the waste of the abundant computing data.

In terms of the application of AMPs, although these novel AMPs have excellent antimicrobial capacity confirmed by a series of in vitro assays, especially in fighting against multidrug-resistant superbugs, there are still multiple challenges in the clinical translation process, such as their inherent limitations and complex drug-tissue relationships. The major challenges are as follows:

1. Poor stability: The stability of AMPs is of great concern. The short half-life of many AMPs has been a hurdle toward their clinical use. For topical application, AMPs are susceptible to damage by environmental (e.g., oxidation, hydrolysis, photolysis) and wound-associated factors (e.g., high pH, protein hydrolysis).
2. Weak activity: Although most AMPs exhibit strong in vitro activity, the in vivo antimicrobial activity may be greatly diminished because of the complexity of the microenvironment, such as the influence of various proteases and dynamic changes in pH.
3. Potential toxicity and unexpected side effects: If used in the clinic, AMPs that interact with cell membranes or bind to receptors are potentially toxic and susceptible to adverse effects, such as cytotoxicity and inflammatory reactions.
4. Unclear structure-tissue exposure/selectivity-activity relationship (STR): Current studies have focused on the identification and design of novel AMPs. At the same time, there is a lack of examination of the STR of AMPs in diseased/normal tissues, which may provide inaccurate information for the selection of drug candidates and affect the clinical dose/efficacy/toxicity balance. The mechanisms of how AMPs exert their biological effects in the response to pathogens inside the body and their dose, duration, and type of effectiveness require further study.
5. High research and development (R&D) costs: Researching AMPs for clinical use requires significant investment, as does developing other antibiotics. Because of the increasing prevalence of antibiotic resistance, the high investment in R&D, and the low conversion rate of AMPs, the economic problems of their development cannot be ignored.

Despite these challenges, there is great potential to utilize the vast amount of genomic information accumulated over the past few years, the databases that have been created, the development of multiple computational platforms, and other resources, and this potential will continue to grow. More and more novel AMPs with incredible antimicrobial activity are expected to enter clinical trials gradually.

## Conclusions and Perspectives

The emergence of antimicrobial resistance (AMR) remains a serious threat and requires urgent action. New antibiotics or their alternatives are immensely needed to address the antibiotic resistance crisis. Antimicrobial peptides (AMPs) are ubiquitous in all known life forms and play a crucial role in the innate immune response to pathogenic infection. AMPs can be obtained by isolation and purification after natural production from the biosynthesis of clusters of genes, by bioinformatics analysis of homology search, or by modern computational design strategy. Strikingly, machine learning (ML) can go a long way in the discovery of novel and highly active AMPs, offering new opportunities for data-driven peptide design. It provides numerous advantages such as improving drug efficacy, predicting medicinal chemistry, and reducing the total time and cost of drug development. Although most AMPs are small (usually less than 100 amino acids), amphiphilic, and cationic, they vary widely in sequence and structure and have significant antimicrobial activities. Moreover, due to their unique action mechanisms, the ability of pathogenic bacteria to develop resistance to them is limited. Therefore, AMPs emerge as effective alternatives to existing antibiotics, with great potential for clinical applications. Although many challenges remain, AMPs are promising in addressing antibiotic resistance. In future studies, given that AMPs are composed of amino acids with short sequences, the flexible use of various directed evolution approaches, chemical modification, and the introduction of non-canonical amino acids may be able to substantially improve their antimicrobial activity and stability, reduce their potential biotoxicity, and the development of drug resistance. Second, the combination of robotics and artificial intelligence (AI) would automate the entire process of screening new candidate AMPs, peptide synthesis, and experimental validation in multiple parallel analyses and applied in an iterative and large-scale fashion. Third, most current studies are performed in vitro, whereas in vivo studies of AMPs should receive more attention. Fourth, highly feasible methods should be further developed to study synergistic interactions between AMPs and conventional antibiotics to salvage drugs that are currently failing due to the development of resistance. Suppose we deepen our insights into the evolutionary diversity of AMPs and their activities in the context of synergistic use with current antibiotics and further explore the evolution of resistance generation. In that case, we may be able to avert a crisis in AMPs like the current crisis in antibiotic resistance.

## Data Availability

No datasets were generated or analysed during the current study.
